# Scolicidal Agents in Hydatid Cyst Surgery

**DOI:** 10.1155/1998/78170

**Published:** 1998

**Authors:** H. Besim, K. Karayalçin, O. Hamamci, Ç. Güngör, A. Korkmaz

**Affiliations:** 1 6th Department of Surgery Ankara Numune Hospital Ankara Turkey; 2 Department of Parasitology Ankara University Faculty of Medicine Ankara Turkey

## Abstract

Injecting scolicidal solutions into the hydatid cyst and packing the operative field with sponges soaked in scolicidal agents have been used to avoid dissemination of the parasite during surgery. In the first part of this *invitro* study, we tried to determine the scolicidal property of various agents in different concentrations and exposure times. In the second part, we tested whether sponges soaked in different type and concentrations of scolicidal agents have any role beyond being a mechanical barrier. 20% saline, 3% hydrogen peroxide, 1.5% cetrimide-0.15% chlorhexidine (10% Savlon®), 95% ethyl alcohol, 10% polyvinylpirrolidone-iodine (Betadine®) and their further dilutions were used in this study. Protoscoleces were obtained from the cyst containing livers of the sheep and viability was determined with dye-uptake (0.1% Eosin) and flame cell activity. Savlon® was found to be the least concentration dependent scolicidal agent among those studied. Scoleces sprayed on sponges soaked in 20% saline, 95% ethyl alcohol, Betadine® and 3% hydrogen peroxide were killed after 15 minutes. 3% and 10% saline and normal saline were ineffective. Sponges work not only as a mechanical barrier but also as a chemical one if the agent is chosen correctly. In purely cystic hydatid liver disease, the risk of dissemination of the cyst contents can be avoided by injection of a potent scolicidal agent such as Savlon®.


*HPB Surgery*, 1998, Vol. 10, pp. 347–351
Reprints available directly from the publisher
Photocopying permitted by license only

© 1998 OPA (Overseas Publishers Association)
Amsterdam B.V. Published under license
under the Harwood Academic Publishers imprint,
part of The Gordon and Breach Publishing Group.
Printed in India.

### Scolicidal Agents in Hydatid Cyst Surgery

H. BESIM[a], K. KARAYALÇIN[a], O. HAMAMCI[a], Ç. GÜNGÖR[b] and A. KORKMAZ[a]

[a]*6th Department of Surgery, Ankara Numune Hospital, Ankara, Turkey;* [b]*Department of Parasitology, Ankara University Faculty of Medicine, Ankara, Turkey*

*(Received 7 November 1996; In final form 8 May 1997)*

**Injecting scolicidal solutions into the hydatid cyst and packing the operative field with sponges soaked in scolicidal agents have been used to avoid dissemination of the parasite during surgery. In the first part of this *invitro* study, we tried to determine the scolicidal property of various agents in different concentrations and exposure times. In the second part, we tested whether sponges soaked in different type and concentrations of scolicidal agents have any role beyond being a mechanical barrier. 20% saline, 3% hydrogen peroxide, 1.5% cetrimide-0.15% chlorhexidine (10% Savlon®), 95% ethyl alcohol, 10% polyvinylpirrolidone-iodine (Betadine®) and their further dilutions were used in this study. Protoscoleces were obtained from the cyst containing livers of the sheep and viability was determined with dye-uptake (0.1% Eosin) and flame cell activity. Savlon® was found to be the least concentration dependent scolicidal agent among those studied. Scoleces sprayed on sponges soaked in 20% saline, 95% ethyl alcohol, Betadine® and 3% hydrogen peroxide were killed after 15 minutes. 3% and 10% saline and normal saline were ineffective. Sponges work not only as a mechanical barrier but also as a chemical one if the agent is chosen correctly. In purely cystic hydatid liver disease, the risk of dissemination of the cyst contents can be avoided by injection of a potent scolicidal agent such as Savlon®.**

*Keywords:* Hydatidosis, scolicidal agents

### INTRODUCTION

Surgery is the treatment of choice for hydatid cyst of the liver since the results of medical and percutaneous treatment are still controversial. In the surgical management of this disease, neutralization of the parasite, evacuation of the cyst and the management of the residual cavity are the principal steps. Prevention of spillage into the peritoneal cavity and wound edges is very important. Injecting a scolicidal agent into the unopened cyst and walling off the operative field with sponges soaked in a scolicidal agent are the two most commonly employed measures although the effectiveness of these measures is not confirmed.

Formalin, hypertonic saline, cetrimide, chlorhexidine, hydrogen peroxide and ethyl alcohol are some of the compounds used as scolicidal. All are concentration dependent and their degree of dilution in the cyst contents is quite unpredictable.

In this *invitro* study, we tried to find out the scolicidal effects of various agents in different

Correspondence to: Hasan Besim MD., Altay Sok. 17/4, Ileri Mah., 06600, Ankara, Turkey.

concentrations and exposure times. In the second part of the study, we tried to check if scoleces sprayed on sponges soaked in different type and concentrations of scolicidal agents could survive after 15 minutes.

The main aim of this study is to determine in an *invitro* basis if the practices of injecting scolicidal agents into the cyst and walling off the operative field with packs soaked in scolicidal agents are likely to achieve the effect for which they are performed.

### MATERIALS AND METHODS

Protoscoleces of *Echinococcus granulosus* were obtained from the cyst containing livers of the sheep slaughtered at a local slaughterhouse in Ankara. Protocoleces were removed from the cysts by aseptic techniques as previously described by Smyth [1].

The material was allowed to settle in a sterile bottle and the supernatant was removed. The remaining sediment contained thousands of protoscoleces. The viability of this suspension was confirmed prior to the experiments. The viability throughout this study was determined by flame cell activity and vital staining with 0.1% eosin. Viable scoleces show flame cell activity and do not take up the dye [2].

The scolicidal agents examined were 10% polyvinylpirrolidone-iodine (Betadine®), 3% hydrogen peroxide, 95% ethyl alcohol, 1.5% cetrimide – 0.15% chlorhexidine (10% Savlon®) and 20% saline, and their further dilutions. The first concentration was generally the one available commercially. The second and the third concentrations were 50% and 10% of the original solutions diluted with sterile distilled water. This was simulating the dilution within the cyst contents.

Two milliliters of each scolicidal solution were placed in test tubes. A drop of protoscolex rich sediment was added to each tube and mixed gently. Following 5 and 10 minutes of exposure, the upper parts of the solutions were removed with a pipette avoiding settled protoscoleces. Then remaining settled protoscoleces were rinsed three times in phosphate buffered saline (PBS) and examined microscopically for viability.

In the second part of the experiment, small (1×1 cm) pieces of sponge were cut and soaked in 20%, 10% and 3% saline, 3% hydrogen peroxide, Betadine®, 95% ethyl alcohol, 10% Savlon® and 0.9% saline as control. A drop of protoscolex rich sediment was sprayed on each sponge and after 15 minutes, sponges were put into different test tubes filled with PBS and shaken vigorously. After taking the sponges out, the remaining solutions were slowly centrifugated, the sediments were placed on slides and examined microscopically for viability.

### RESULTS

The results of the first part of this study was shown in Table I. Betadine® was the first scolicidal solution to be studied. Its undiluted and 50% diluted forms were both effective in terms of killing the protoscoleces but when the concentration was lowered to 10% (1% polyvinylpirrolidone-iodine), the protoscoleces were found to be alive after 5 or 10 minute exposures.

Savlon® solution was very effective in all concentrations up to 1% Savlon® and the morphology of the protoscoleces was distorted after coming in contact with this substance. A further dilution of 0.1% Savlon® (0.015% cetrimide – 0.0015% chlorhexidine) was prepared and this was also scolicidal in 5 and 10 minutes exposures.

3% hydrogen peroxide was effective in both undiluted and 50% diluted forms but no scolicidal property can be shown with 0.3% hydrogen peroxide even at the 10 minutes exposure.

95% ethyl alcohol was found to be effective only in undiluted forms. Further dilutions of 47% and 9.5% ethyl alcohol were all ineffective

TABLE I Scolicidal effects of selected agents in various dilutions and exposure times

| Exposure Time | 5 Minutes | | 10 Minutes | |
|---|---|---|---|---|
| Scolicidal Agent | Test | Viability | Test | Viability |
| Betadine® | E(+) FCA(−) | Dead | E(+) FCA(−) | Dead |
| 50% Betadine® | E(+) FCA(−) | Dead | E(+) FCA(−) | Dead |
| 10% Betadine® | E(−) FCA(+) | Live | E(−) FCA(+) | Live |
| 10% Savlon® | E(+) FCA(−) | Dead | E(+) FCA(−) | Dead |
| 5% Savlon® | E(+) FCA(−) | Dead | E(+) FCA(−) | Dead |
| 1% Savlon® | E(+) FCA(−) | Dead | E(+) FCA(−) | Dead |
| 0.1% Savlon® | E(+) FCA(−) | Dead | E(+) FCA(−) | Dead |
| 3% Hydrogen Peroxide | E(+) FCA(−) | Dead | E(+) FCA(−) | Dead |
| 1.5% Hydrogen Peroxide | E(+) FCA(−) | Dead | E(+) FCA(−) | Dead |
| 0.3% Hydrogen Peroxide | E(−) FCA(+) | Live | E(−) FCA(+) | Live |
| 95% Ethyl Alcohol | E(+) FCA(−) | Dead | E(+) FCA(−) | Dead |
| 47.5% Ethyl Alcohol | E(−) FCA(+) | Live | E(−) FCA(+) | Live |
| 9.5% Ethyl Alcohol | E(−) FCA(+) | Live | E(−) FCA(+) | Live |
| 20% Saline | E(+) FCA(−) | Dead | E(+) FCA(−) | Dead |
| 10% Saline | E(−) FCA(+) | Live | E(+) FCA(−) | Dead |
| 2% Saline | E(−) FCA(+) | Live | E(−) FCA(+) | Live |

E: 0.1% Eosin, FCA: Flame cell activity, Betadine®: 10% Polyvinylpirrolidone-iodine, Savlon®: 15% cetrimide-1.5% chlorhexidine.

as a scolicidal agent in both 5 or 10 minutes exposures.

20% saline is a widely used scolicidal agent. Undiluted form of this substance killed the protoscoleces in both 5 and 10 minutes of exposures. 10% saline could not kill the protoscoleces in 5 minutes but when we prolong the exposure time, the protoscoleces were killed at 10 minutes. 2% saline could not show any lethal effect on the protoscoleces.

The results of the second part of this study; the effectiveness of sponges soaked in various scolicidal agents, are summarized in Table II. Protoscoleces sprayed on to different sponges soaked in Betadine®, 20% saline, 3% hydrogen peroxide, 95% ethyl alcohol, and 10% Savlon® were inactivated after 15 minutes. But sponges soaked in 10% saline, 3% saline and 0.9% saline were not effective and protoscoleces were alive after 15 minutes exposure.

### DISCUSSION

Although surgery is considered the treatment of choice for hydatid disease of the liver, controversies still exist regarding the preferred operative technique, management of the residual cavity and the use of scolicidal agents.

It has been traditional to inject scolicidal agents into the unopened hydatid cyst because of the risk of spillage into the peritoneal cavity leading to recurrent disease. Cyst fluid contains thousands of protoscoleces and each one has the potential to grow into a new hydatid cyst.

Among the various scolicidal agents advocated in the past, formalin was the first and most frequently used agent. Despite its effectiveness, it is no longer used because of the associated toxicity [3]. Ethyl alcohol is the scolicidal agent that is usually preferred for ultrasonic-guided percutaneous aspiration, injection and reaspiration (PAIR) of hydatid cysts [4, 5]. Unfortunately, it can cause caustic damage to the epithelium of communicating bile ducts leading to sclerosing cholangitis and it is strongly concentration dependent [6] (Tab. I). Hydrogen peroxide has not gained wide acceptance because of low efficacy and complications [7]. Betadine® is a disinfectant that is used as a scolicidal agent by many surgeons but PVP (*polyvinylpirrolidone*) storage disease, renal shutdown, sterile peritonitis and sclerosing serositis are the associated complications and its use is

TABLE II The viability of scoleces sprayed on scolicidal agent soaked sponges

| Scolicidal Agent | Scolex Viability After 15 Minute Exposure | |
|---|---|---|
| Betadine® | 0.1% Eosin(+), FCA(−) | Dead |
| 20% Saline | 0.1% Eosin(+), FCA(−) | Dead |
| 10% Saline | 0.1% Eosin(−), FCA(+) | Live |
| 3% Saline | 0.1% Eosin(−), FCA(+) | Live |
| 3% Hydrogen Peroxide | 0.1% Eosin(+), FCA(−) | Dead |
| 95% Ethyl Alcohol | 0.1% Eosin(+), FCA(−) | Dead |
| 10% Savlon® | 0.1% Eosin(+), FCA(−) | Dead |
| 0.9% Saline(control) | 0.1% Eosin(−), FCA(+) | Live |

E: 0.1% Eosin, FCA: Flame cell activity, Betadine®: 10% Polyvinylpirrolidone-iodine, Savlon®: 15% cetrimide-1.5% chlorhexidine.

restricted to preoperative local antisepsis of intact adult skin [8].

Hypertonic saline and cetrimide have become the scolicidal agents of choice over the past years. Although it was demonstrated that 5% saline has no effect on scoleces, many surgeons have recommended the use of 3% saline [9, 10]. Our results support the findings of Saidi [11] as no scolicidal effect can be shown with a concentration of less than 10% saline at 5 minutes. Lowest concentration of *saline* should be 20% and it should not be used in patients who have cysts communicating with biliary tree because of the danger of causing caustic sclerosing cholangitis [6].

Cetrimide is a potent disinfectant and effective scolicidal agent [12]. Low concentrations of cetrimide (0.1 – 0.5%) have been used by many surgeons [12 – 14]. We used cetrimide with chlorhexidine that was also recommended as a scolicidal agent [15] because this combination is a widely available disinfectant solution named as Savlon®. The results of this study showed that Savlon® is a very potent scolicidal agent even at very low concentrations that makes it the scolicidal agent of choice in the situations where it is hard to anticipate the volume of the cyst and adjust for dilution of the scolicidal agent. Although cetrimide is effective in very low concentrations, it is not devoid of complications. Gilchrist [16] reported three cases of sclerosing peritonitis after peritoneal washout to prevent secondary hydatidosis. Metabolic acidosis and methemoglobinemia were the two other reported complications due to cetrimide installation into hydatid cysts [17, 18]. The effect of cetrimide on the biliary duct epithelium has not so far been studied which makes its use questionable in the cases with cysts communicating with the bile ducts.

Walling off the surgical field with laparotomy sponges or packs soaked in scolicidal agents is an effective and logical means of using scolicidal agents if the agent is chosen correctly.

Although it is a common practice to inject scolicidal agents into hydatid cysts, lack of objective evidence about the efficacy and the presence of toxicity associated with the scolicidal agents have led many surgeons to abandon this routine step in the operative management of hydatid cysts [18 – 20]. Particularly in multivesicular cysts, daughter cysts will not be influenced as it is impossible to puncture each of them. However in purely cystic hydatid liver disease, the risk of dissemination of the cyst contents can be avoided by injection of a potent scolicidal agent such as Savlon®.

### *References*

[1] Smyth, J. D. (1962). Studies on tapeworm physiology: Axenic cultivation of the hydatid organism, echinococcus granulosus; establishment of a basic technique. *Parasitology*, **52**, 441 – 457.

[2] Smyth, J. D. and Barret, N. J. (1980). Procedures for testing the viability of human hydatid cyst following surgical removal, especially after chemotherapy. *Transactions of the Royal Society of Tropical Medicine and Hygiene*, **74**, 649 – 652.

[3] Aggarwal, A. R. and Garg, R. L. (1983). Formalin toxicity in hydatid liver disease. *Anaesthesia*, **38**, 662–665.
[4] Giorgio, A., Tarantino, L., Francica, G., Mariniello, N., Aloisio, T., Soscia, E. and Pierri, G. (1992). Unilocular hydatid liver cyst: Treatment with US-guided, double percutaneous aspiration and alcohol injection. *Radiology*, **184**, 705–710.
[5] Filice, C., Pirola, F., Brunetti, E., Dughetti, S., Strosselli, M. and Scotti-Foglieni, C. (1990). A new therapeutic approach for hydatid liver cyst. Aspiration and alcohol injection under sonographic guidance. *Gastroenterology*, **98**, 1365–1368.
[6] Castellano, G., Moreno-Sanchez, D., Gutierrez, J., Moreno-Gonzalez, E., Colina, F. and Solis, Herruzo, J. A. (1994). Caustic sclerosing cholangitis. Report of four cases and a cumulative review of the literature. *Hepato Gastroenterology*, **41**, 458–470.
[7] Magistrelli, P., Masetti, R., Coppola, R., Messia, A., Nuzzo, G. and Picciocchi, A. (1991). Surgical treatment of hydatid disease of the liver. A 20-year experience. *Archieves of Surgery*, **126**, 518–523.
[8] LeVeen, H. H., LeVeen, F. R. and LeVeen, G. E. (1993). The Mythology of Povidone-iodine and the Development of Self-sterilizing Plastics. *Surgery, Gynecology and Obstetrics*, **176**, 183–189.
[9] Little, J. M. and Deane, S. A. (1988). Hydatid Disease. In Surgery of the Liver and Biliary Tract, edited by Blumgart L. H., pp. 955–967. Edinburgh: Churchill Livingstone.
[10] Milicevic, M. (1994). Hydatid Disease. In Surgery of the Liver and Biliary Tract, edited by Blumgart L. H., pp. 1121–1150. Edinburgh: Churchill Livingstone.
[11] Saidi, F. (1976). *Surgery of Hydatid Disease*, pp. 31–59. Philadelphia: W. B. Saunders Company.
[12] Ahrari, H. (1978). Use of cetremide in the surgery of hydatid cysts. *Bulletin de la Societe de Pathologie Exotique et de ses Filiales*, **71**, 90–94.
[13] Frayha, G. J., Bikhazi, K. J. and Kachachi, T. A. (1981). Treatment of hydatid cysts (Echinococcus granulosus) by cetrimide (R). *Transactions of the Royal Society of Tropical Medicine and Hygiene*, **75**, 447–450.
[14] Schaefer, J. W. and Khan, M. Y. (1991). Echinococcosis (hydatid disease): lessons from experience with 59 patients. *Reviews of Infectious Diseases*, **13**, 243–247.
[15] Langer, C. J., Rose, B. D., Keystone, S. J., Taylor, R. B. and Langer, B. (1984). Diagnosis and management of hydatid disease of the liver. A 15-year North American experience. *Annals of Surgery*, **199**, 412–417.
[16] Gilchrist, D. S. (1979). Chemical peritonitis after cetrimide washout in hydatid-cyst surgery. *Lancet*, **ii**, 1374.
[17] Momblano, P., Pradere, B., Jarrige, N., Concina, D. and Bloom, E. (1984). Metabolic acidosis induced by cetrimonium bromide. *Lancet*, **ii**, 1045.
[18] Baraka, A., Wakid, N. and Yamout, F. (1980). Methemoglobinemia during surgical excision of hydatid cyst. *Middle East Journal of Anaesthesia*, **5**, 509–513.
[19] Belghiti, J., Benhamou, J. P., Houry, S., Grenier, P., Huguier, M. and Fékété, F. (1986). Caustic Sclerosing Cholangitis. *Archives of Surgery*, **121**, 1162–1165.
[20] Prasad, J., Bellamy, P. and Stubbs, R. S. (1991). Instillation of scolicidal agents into hepatic hydatid cysts: can it longer be justified? *New Zealand Medical Journal*, **104**, 336–337.

### COMMENTARY ON PAPER, "SCOLICIDAL AGENTS IN HYDATID CYST SURGERY", BY BESIM *et al.*

Injecting scolicidal agents into hydatid cysts prior to their opening, and packing off the operative field with packs soaked in scolicidal agents have been standard aspects of the surgical management of hydatid disease for many years. However there is little clear evidence attesting the value of these manoevers and the paper by Dr Besim and colleagues provides compelling evidence pointing to the futility of injecting the commonly used scolicidal agents into the cysts, with the possible exception of Savlon. This knowledge coupled with the accummulated evidence indicating that biliary stricturing may follow the instillation of scolicidal agents into cysts provides compelling reasons for the practice to be abandoned.

The use of one months pre-operative treatment with albendazole can be recommended because it results in substantial killing of cyst contents and leads to relative collapse of the cysts, which may therefore be opened without spurting of cyst fluid to contaminate the surgical field and predispose to recurrent hydatid disease. There clearly remains a place for the use of packs, soaked in an appropriate concentration of scolicidal agent, for both walling off the operative field and laying within cyst cavities after these have been substantially evacuated. The authors are to be commended for their study.

Richard S. Stubbs MD FRCS FRACS
The Wakefield Clinic, Wellington
New Zealand

